# A mammalian messenger RNA sex determination method from humpback whale (*Megaptera novaeangliae*) blubber biopsies

**DOI:** 10.1098/rsos.220556

**Published:** 2022-08-24

**Authors:** Jacob M. J. Linsky, Rebecca A. Dunlop, Michael J. Noad, Lee A. McMichael

**Affiliations:** ^1^ School of Biological Sciences The University of Queensland, St Lucia, Queensland 4072, Australia; ^2^ Centre for Marine Science, The University of Queensland, St Lucia, Queensland 4072, Australia; ^3^ School of Veterinary Science, The University of Queensland, Gatton, Queensland 4343, Australia

**Keywords:** sex determination, *XIST*, humpback, whale, RNA, gene expression

## Abstract

The large size of free-ranging mysticetes, such as humpback whales (*Megaptera novaeangliae*), make capture and release health assessments unfeasible for conservation research. However, individual energetic condition or reproductive health may be assessed from the gene expression of remotely biopsied tissue. To do this, researchers must reliably extract RNA and interpret gene expression measurements within the context of an individual's sex. Here, we outline an RNA extraction protocol from blubber tissue and describe a novel mammalian RNA sex determination method. Our method consists of a duplex reverse transcription-quantitative (real-time) polymerase chain reaction (RT-qPCR) with primer sets for a control gene (*ACTB*) and the X-chromosome inactivation gene (*XIST*). Products of each RT-qPCR had distinct melting temperature profiles based on the presence (female) or absence (male) of the *XIST* transcript. Using high-resolution melt analysis, reactions were sorted into one of two clusters (male/female) based on their melting profiles. We validated the *XIST* method by comparing results with a standard DNA-based method. With adequate quantities of RNA (minimum of approx. 9 ng µl^−1^), the *XIST* sex determination method shows 100% agreement with traditional DNA sex determination. Using the *XIST* method, future cetacean health studies can interpret gene expression within the context of an individual's sex, all from a single extraction.

## 1. Introduction

Many baleen whale, or mysticete, species were driven to near extinction because of commercial whaling by the mid-twentieth century [1]. Since that time, the recovery of mysticete populations has been varied, ranging from the still critically endangered North Atlantic right whale (*Eubalaena glacialis*), to the slowly recovering blue whale (*Balaenoptera musculus*), to the rapidly recovering humpback whale (*Megaptera novaeangliae*, [2–6]). The different trajectories of these post-whaling mysticete population recoveries are linked to individual health parameters, such as body condition and reproductive productivity [7]. Monitoring of these parameters is highly desirable for managing mystecete recovery, However, much of the baseline information required to do this is missing for mystecete species. To fill this information gap, new cost-effective methods to measure the health of free-ranging whales are required.

One promising health monitoring tool is real-time (quantitative) polymerase chain reaction (qPCR), which can measure gene expression from biopsied tissue samples. qPCR has a wide variety of potential animal health diagnostic applications, including pathogen detection [8], measurement of endocrine function [9] and measurement of the impacts pollutants have on an individual's gene expression [10], meaning it has enormous potential to be used as a tool for monitoring cetacean health. The expression of specific genes, measured from messenger RNA (mRNA), can also be linked to biologically significant changes in animal physiology and behaviour at time scales ranging from diurnal to seasonal, to over a lifetime [11]. While these seasonal transcriptome changes have been described in some cetacean species, such as the common bottlenose dolphin (*Tursiops truncatus*, [12]), for most cetaceans this critical baseline information is yet to be determined.

Part of the reason for the lack of baseline health data is the limited tissue available for free-ranging cetacean studies. The expression of specific genes varies between tissues [13]. For example, genes related to fat metabolism show much higher expression in the brain than in the heart, regardless of an individual's circumstances [14]. Thus, for a tissue to be used to monitor a specific health feature, it must express genes related to that feature. In humpback whales, along with other cetaceans, gene expression studies are primarily limited to remotely biopsied samples consisting of skin and blubber, a thick layer of subcutaneous vascularized adipose tissue. Of these two tissues, blubber is especially desirable as a potential reservoir of health information owing to its wide range of roles in energy regulation, immune function, reproduction and thermoregulation [15]. However, some previous gene expression studies in whale species have reported challenges with the yield and integrity of RNA extractions from blubber tissue, probably because of the high lipid content and low quantities of RNA [12,16,17]. Thus, a simple and repeatable blubber RNA extraction protocol is an essential prerequisite for cetacean gene expression studies, particularly those that are investigating parameters that may be measurable in blubber tissue rather than in skin, such as energy reserves. Once extracted, knowledge of an individual's baseline gene expression is required if RNA is to be used as a health assessment tool. However, gene expression is dynamic, and contextual information about sampled individuals is needed to understand typical versus atypical expression levels of specific genes [18].

One essential piece of contextual information is an individual's sex as baseline expression of many genes can vary between sexes [19]. Humpback whales, and most other mysticetes, are capital breeders, meaning that fasting females may have extreme differences in the costs and physiological requirements of reproduction compared to males [20]. These differences in reproductive demands may lead to increased sexual differences in the expression of certain genes. However, for humpback whales and other cetacean studies, sex is not easily visually determined [21,22]. Instead, sex is often determined post-sampling via molecular analysis of biopsied tissue.

Molecular methods for mammalian sex determination typically use extracted genomic DNA (gDNA) to identify the presence or absence of genes related to the Y-chromosome. The most common applications use traditional PCR and specific primer sets to amplify the zinc finger protein X or Y-linked (*ZFX/ZFY*) sequences [23–25] or ‘sex determining region Y’ (*SRY*) sequences [26–28]. If a researcher, however, wants to study gene expression, RNA is required. For gene expression studies, it would be more efficient, both in terms of costs and tissue usage, to determine sex using the same RNA extracted for the study. Unfortunately, established methods of sex determination from gDNA may not readily be adapted to mRNA studies. This is because genes that uniquely identify the Y-chromosome (i.e. lack X homologues) show limited expression outside of the testis [29]. Ideally, the target sequence in a sex determination test should be expressed in all an animal's tissues and clearly distinguish (i.e. test presence versus absence) between the sexes for simplicity of analysis.

One RNA-based mechanism that fits these criteria relies on the ‘X-chromosome inactivation gene’ (*XIST*). In every female cell, one of the two X chromosomes is ‘silenced’, meaning that the expression of genes on that chromosome is dramatically reduced [30]. This process is regulated by the *XIST* gene in all mammalian cells that contain more than one X chromosome [30], meaning that the presence or absence of *XIST* mRNA in any somatic tissue can be used to identify females, or males, respectively. *XIST* mRNA presence has been used to identify fetal sex in the early stages of pregnancy for humans [31] and some domestic animals [32]. These results highlight the potential for an *XIST*-based sex determination test to function for mRNA as the *SRY* method functions for DNA. Like traditional PCR used in the *SRY* method, reverse transcription-quantitative (real-time) polymerase chain reaction (RT-qPCR) can run in multiplex [33]. This allows for reduced costs as experimental and control genes can be combined in a single reaction. Unlike traditional PCR, however, the presence of different RT-qPCR products in a reaction can be easily and efficiently identified from their unique melting temperatures using high-resolution melt (HRM) analysis [34,35]. This means that the entire sex determination process can be accomplished with one piece of equipment, a qPCR machine, making for an efficient method of sex determination for gene expression studies.

Humpback whales are among the most successful recoveries of the once commercially hunted mysticete species [36]. Like most mysticetes, humpback whales have a critical migratory breeding period during which little is known about individual health. However, the abundance of the eastern Australian humpback whale, and the tendency of this population to move along an accessible coastal migration corridor, makes this population ideal for developing novel cetacean health monitoring techniques. This study addresses two prerequisites for the use of gene expression in cetacean health studies. First, we aim to provide a reliable extraction protocol for RNA from the blubber of free-ranging humpback whales off eastern Australia. Second, we aim to develop a novel RNA sex determination method (*XIST* method) using RT-qPCR and validate the novel method by comparing results to the standard *SRY* sex determination method. Together these aims provide a reliable and efficient basis for future health studies by allowing gene expression to be interpreted within the context of an animal's sex from a single extraction.

## 2. Methods

### 2.1. Sample collection

Biopsies (*n* = 104) were collected from free-ranging humpback whales off the coast of North Stradbroke Island (Minjerribah) and Peregian Beach, Queensland, Australia, during 2020 (*n* = 35) and 2021 (*n* = 69) annual migration to and from their breeding grounds in the Great Barrier Reef (June to November). The samples were collected from live animals using a Paxarm MK24C remote biopsy system. Biopsies were typically taken from the flank (below the dorsal area) or peduncle of the animal using a floating biopsy dart with a cutting head measuring 7 mm in diameter and 40 mm in length. Usually, biopsies contained both skin and blubber. However, sometimes the dart failed to recover a full sample and only skin was present. For the 2020 season, biopsy darts were collected from the ocean's surface, placed into resealable plastic bags and stored on ice for 2–8 h while onboard the vessel, then transferred to −80°C onshore. RNA was extracted 7–10 months after the sample collection. During the 2021 season, subsamples of approximately 50 mg were cut from about 35 mm into the blubber layer immediately after collection. These subsamples were stored in 1 ml of Qiagen RNAprotect (product number: 76104) and then placed on ice on-board the vessel. Upon returning to shore, 2021 samples were stored identically to the 2020 samples at −80°C until the RNA extraction process. For the 2021 samples, extraction occurred three to six months after collection. All samples were accompanied by notes on the animal's behaviour and calf presence (as an additional control to identify females), and identification photographs of the dorsal fin and underside of the fluke when possible.

### 2.2. RNA extraction and complementary DNA synthesis

RNA was extracted using the Qiagen RNeasy Universal Mini kit (product number: 73404). For the 2020 season, the first four samples were extracted according to the manufacturer's kit protocol. The remaining 31 samples from 2020 and all samples from 2021 underwent the extraction process with alterations to the manufacturer's kit protocol as follows (see the electronic supplementary material for full protocol). Approximately 50 mg of each tissue was ground in liquid nitrogen with a mortar and pestle. To promote the lysis and homogenization of the high lipid content blubber cells, a 2% volume of Triton X-100 detergent was added to the kit-provided lysis buffer. Following the evaporation of the nitrogen, the modified lysis buffer mix was added to the mortar and the sample was homogenized manually. The sample was then incubated for 30 min at room temperature, with occasional inversion to maximize contact between the sample and the lysis buffer. Both the kit-provided gDNA digestion and an on-column DNA digestion using the Qiagen RNase-free DNase set (product number: 79254) were performed according to protocol. RNA was collected with a single 50 µl H_2_O elution. Following extraction, RNA yields (ng µl^−1^) were measured using a NanoDrop 1000 spectrophotometer (Thermo Fisher Scientific).

Complementary DNA (cDNA) synthesis was performed using the Bioline Tetro cDNA synthesis kit and random hexamers (product number: BIO-65042) according to manufacturer protocols. The presence of cDNA in the reaction was confirmed (approx. 200 ng µl^−1^ per sample) using a NanoDrop 1000 spectrophotometer.

### 2.3. Primer design

Primers were designed using the GenBank primer designing tool [37] and were supplied by Sigma Oligos. A control transcript, *β-actin* (*ACTB*), was identified using an existing accession from the MegNov1 assembly (GCA_004329385.1). The *XIST* transcript was located using the basic local alignment search tool (BLAST) to match *XIST* sequences of the closely related domestic ox (*Bos taurus*, XR_001495596.2) with the MegNov 1 humpback whale assembly. Primers were designed for the most likely matched region in the humpback whale genome: RYZJ01002456.1 (1049146–1051170). All primers were then checked for specificity using the UCSC in Silico PCR search tool against the closest available genome, the northern minke whale (*Balaenoptera acutorostrata*). The primers *ACTB*_mn01F (forward sequence: 5′ AAGATCCTCACGGAGCGTGG 3′) and *ACTB*_mn01R (reverse sequence: 5′ TGATCACCTGACCATCGGGC 3′) were used to amplify the *ACTB* control transcript. The primers *XIST*_mn01F (forward sequence: 5′ CCGTTACATTCTTGGCGGGC 3′) and *XIST*_mn01R (reverse sequence: 5′ TCCTCCACTAACTGGCTGCG 3′) were used to amplify the *XIST* female-specific transcript. Following initial PCR amplification, amplicons for each primer set were sequenced by the Australian Genome Research Facility (see the electronic supplementary material). Sequences were aligned with existing sequences in the Genbank database using BLAST [38] to confirm that each primer amplified the intended target.

### 2.4. Sex determination via reverse transcription-quantitative polymerase chain reaction

Samples were run in triplicate using a 10^−1^ dilution of the cDNA template. All primers had a final concentration of 0.25 µM each. Thermo Fisher Powerup Syber Green Master Mix (product number: 4368577), primers and template were added (see the electronic supplementary material) to a 96 well plate and run using a Bio-Rad CFX 96 Touch thermocycler. Reactions were run as primer duplexes, containing both the *ACTB* and *XIST* primer sets. The thermocycling profile was 2 min at 50°C and 2 min at 95°C, followed by 45 cycles of 15 s at 95°C, 15 s at 64°C and 60 s at 72°C. A melt curve step to measure the relative fluorescence units (RFUs) with 0.5°C intervals followed the final PCR cycle (see Bio-Rad CFX 96 Touch thermocycler manual for details).

Data from each run were imported into the Bio-Rad Precision Melt Analysis software (product number: 1845015). An HRM analysis was conducted to group the samples into distinct clusters based on their melt signatures. The only inputs for this software are the melt curve data from each RT-qPCR run and user-defined values for melt curve shape sensitivity and melting temperature (*T*_m_). The number of clusters and the assignment of wells to each cluster are automated based on these input parameters. For this analysis, the melt curve shape sensitivity was set to minimum (1%) and the *T*_m_ difference threshold was set to maximum (1°C). These settings were chosen to avoid clustering based on any fine-scale differences in product sequences between individuals (i.e. different alleles or single nucleotide polymorphisms). Instead, the algorithm produced broad clusters related to the presence, or absence, of *XIST* expression. Wells that did not amplify any product were excluded from further analysis. A sample was assigned sex status (male/female) if a majority of that sample's wells (greater than or equal to two-thirds) were in a given cluster.

### 2.5. DNA extraction and conventional polymerase chain reaction validation

DNA was extracted from the skin portion of each sample using the Meridian Bioscience MyTaq Extract-PCR Kit (product number: BIO-21126) according to the manufacturer's protocol. The only variance from the kit protocol was an increase in incubation time from 5 min to 12 min to ensure adequate lysis of the skin cells. Previously established primers for a positive control gene, *ZFX/ZFY* [23], and the Y-chromosome identification gene, *SRY* [26], were applied to a conventional gel-based PCR according to an established sex determination protocol for placental mammals [28]. Each sample was assigned a sex based on the presence of one (female) or two (male) bands in the gel.

## 3. Results

### 3.1. RNA extraction

**Figure 1.**
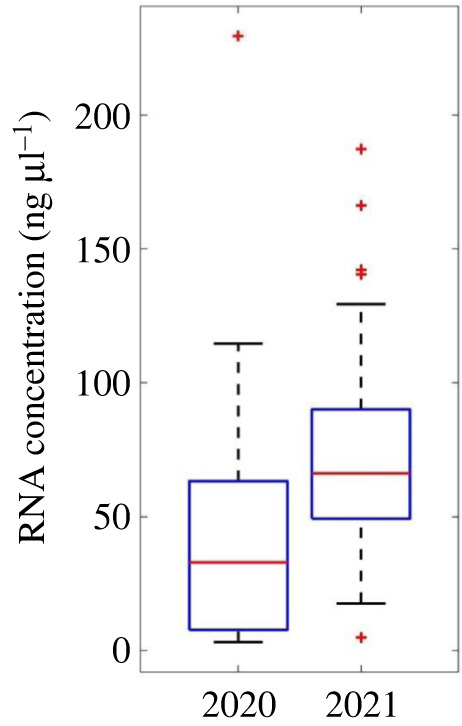
Box plot of RNA extraction yields between samples with (2021) and without (2020) storage in an RNA preserving regent. The central red line of each plot represents the median value, while the box contains the 25th–75th percentile of the data. Outliers are represented by red crosses.

RNA extraction yields varied between 3.1 and 229.4 ng µl^−1^. The initial subset of four samples from 2020 showed a maximum RNA yield of 7.7 µg ml^−1^, with a mean yield of 5.6 ng µl^−1^. The improved TritonX-100 protocol resulted in increased extraction efficiency, producing a mean yield of 55.0 ng µl^−1^. Samples from 2021 that were stored in RNAprotect regent and extracted using the optimized protocol showed improved mean yields of 71.4 ng µl^−1^ (figure 1). RNA yields from the 2020 samples show that biologically relevant quantities of RNA can be obtained from samples left on ice for 4–8 h if need be. However, the increase in 2021 RNA yields indicates that performance is improved by taking preservative steps for the RNA.

### 3.2. Sex determination

**Figure 2.**
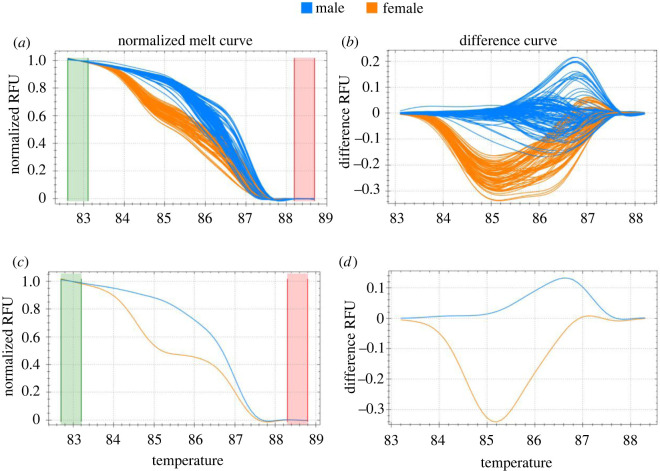
Melt curve and clustering results from HRM analysis. Relative fluorescent units (RFUs) represent the relative quantity of amplified products remaining in a well as temperature increases. (*a*) and (*b*) show all reactions from the optimized 2021 data. (*c*) and (*d*) represent a typical reaction for one male and one female. The normalized melt curves (*a*,*c*) use the green and red shaded areas to represent the pre and post-melt temperatures for data normalization between values of 1 and 0. The difference curve (*b*,*d*) is generated by subtracting each melt curve from a ‘typical’ melt curve in the study. In this case, the software automatically selected an animal from the male group for comparison.

Initial HRM analysis showed that nearly all the reactions belonged to one of two primary groups. The first (male) cluster consisted of 190 wells while the second (female) cluster, consisted of 123. Four reactions (from a total of three individuals) were sorted into outlying clusters. Notably, all four of these reactions occurred in 2020 pilot samples with poor extraction values (3.1–7.7 ng µl^−1^). These wells showed distinct HRM profiles and were excluded from further analysis as they consisted of non-specific products or primer dimers resulting from inadequate templates for cDNA synthesis and RT-qPCR. All samples from 2021 were sorted into one of the two primary clusters (figure 2*a*,*b*).

Known females from field observations of calves were used to identify the cluster that contained females. The other cluster was identified as representing males. After initial identification, several features could be used to distinguish sex clusters. Females showed a steep and significant drop in the difference RFU values at around 84°C, indicating the melting of *XIST* products that are not in male wells. Males were comparatively stable and often showed increased RFU values at around 85.5°C (figure 2*b*,*d*). Additionally, male wells typically showed a smooth theta decay in their normalized melt curve (or only a slight elevation-separating plateau for individuals that were heterozygous for *ACTB* polymorphisms), indicating the decay of a single product (i.e. *ACTB*; figure 2*a*,*c*). Female wells, however, usually showed two RFU elevations in the normalized melt curves, indicating the melting of two separate products (i.e. *ACTB* and *XIST*; figure 2*a*,*c*).

Each sample was assigned a sex based on two or more of its wells fitting into a cluster. Results identified 63 males, 38 females and three inconclusive individuals.

### 3.3. Validation

Sex determination results from the RT-qPCR HRM analysis were compared to results using the *SRY* method from extracted gDNA. Of all the samples (*n* = 104), 100 samples showed agreement between the two methods. The other four samples consisted of the three individuals unidentified using HRM (owing to outside clustering and/or wells split between the two primary clusters) and one female that was misidentified as a male in the HRM analysis.

The four misidentified individuals all had poor RNA extractions in common (3.1–7.7 ng µl^−1^). For the misidentified female, the *XIST* products were probably present, but higher quantification cycle values meant that they could not be identified in the given number of cycles. However, because of the prevalence of off-target products at these low values (shown by the other three samples), the preferred treatment is to repeat the mRNA extraction should there be a yield of less than about 10 ng µl^−1^.

The 2021 sample cohort yielded 100% agreement between *XIST* mRNA and *SRY* gDNA sex determination (*n* = 69). The improved RNA extraction quantities of 2021 samples (figure 1), combined with the absolute agreement between sex determination methods, indicates that with proper storage and optimized extraction, *XIST* presence can serve to reliably determine an individual's sex from mRNA.

## 4. Discussion

For cetacean health studies, gene expression, measured using RT-qPCR, is emerging as a potential reservoir of critical health information. However, for this information to be accessed, RNA must be extracted from lipid-rich adipose tissue, and then expression levels need to be understood within the context of an individual's sex. The overall goal of this study was to develop a robust method of RNA extraction and sex determination from a specific kind of adipose tissue, blubber. Here, we addressed two prerequisites for measuring and interpreting gene expression to monitor the health of humpback whales: RNA extraction from blubber, and the development and validation of a resource-efficient sex determination method using extracted RNA. Our results show that with a few alterations to a commercially available protocol, and optimized sample storage, usable RNA could reliably be extracted from humpback whale blubber. With the extracted RNA, we developed a novel sex determination method (*XIST* method). For efficiency, we designed a duplex PCR reaction that combines both a housekeeping gene (Beta-Actin) and *XIST* using cost-effective reagents. HRM analysis was used to sort individual reactions into one of two distinct clusters representing each sex. Finally, we validated the novel *XIST* sex determination method by comparing results to the commonly used *SRY* sex determination method. For the samples with optimized storage and RNA extraction, we found 100% agreement between the novel (*XIST*) and validation (*SRY*) methods (*n* = 69). This method will allow future studies to measure gene expression within the context of an animal's sex with only a single RNA extraction and an RT-qPCR machine.

The success of our blubber extraction and *XIST* method simplifies the use of blubber mRNA for further applications. Arguably, the application of greatest interest is the use of blubber mRNA to monitor animal health. Adipose tissue, including blubber, is known to play an important role in energy storage, thermoregulation and immune function (see [15,39–41]). These functions make adipose a potential reservoir of health information for any animal. This is especially true for species like marine mammals, where remote biopsies of skin and blubber may be the only source of tissue readily available from living animals. The expression of adipokines is linked to sex-specific differences in humans and some domestic species, indicating that the sex of an animal is essential contextual information to understand adipokine expression in wildlife [42–44]. For studies investigating wildlife health gene expression, the ability to determine sex and measure health parameters from a single mRNA extraction saves resources, labour and time over existing DNA methods. The *XIST* method outlined in this study should, in theory, provide these benefits for most other eutherian species where molecular sex determination is required to interpret gene expression. However, applying the *XIST* method to a novel species certainly requires validation, and may also require protocol adjustments such as specialized primers or changes in the PCR thermocycling profile.

Disagreement between the *SRY* and *XIST* methods was only observed in non-optimally stored samples. In these cases, the performance of the *XIST* sex determination method was most impacted by low RNA yields (i.e. all four misidentified individuals from the 2020 samples shared single-digit ng µl^−1^ RNA concentrations). Because of this, it is strongly recommended that the RNA yield be measured against a pre-determined cut-off. In this study, 9–10 ng µl^−1^ of RNA was the lower limit for reliable detection of *XIST* expression. Samples with low RNA yields may be extracted again using more starting tissue to increase the RNA yield to an acceptable concentration. If a future study was forced to proceed with low RNA yields, one important consideration is that this method could still be used to positively identify some females. In these circumstances, however, the male cluster will probably contain both males and misidentified females. This is because with low RNA yields there is a risk that *XIST* products are present but have not reached the critical mass required for HRM analysis detection while the more abundant *ACTB* products have. These results would produce a melt profile indistinguishable from the male condition where *ACTB* is present and *XIST* is not. Ideally, however, both misidentification and non-specific binding can be avoided through proper sample handling and mRNA extraction (as shown by 100% agreement for 2021 samples).

Another potential source of disagreement between the novel and validation methods used here is XXY aneuploidy, which is also known as Klinefelter syndrome in humans. Although it was not observed in the subjects used for this study, this disorder has been observed in many non-human species, including some cetaceans [45]. It has a comparatively high prevalence in humans, with an estimated 1 in 500 males affected [46]. XXY aneuploidy could be expected to result in positive detection of both *XIST* expression (confirming female) and *SRY* presence (confirming male). This condition probably indicates infertility of the individual thus complicating the results, as the individual cannot fulfil the biological role of a male or a female. For studies interested in this anomaly, however, a combination of the *XIST* and *SRY* sex determination methods may be used to identify the presence of XXY aneuploidy. For such studies, the relative amount of *XIST* expressed could even be explored as a means of identifying the specific number of X chromosomes present in an individual's genome.

One of the benefits of the *XIST* method for gene expression studies is that it reduces costs by preventing the need for additional extraction kits and PCR supplies for DNA-based sex determination. Costs could be reduced further by decreasing reagent volumes or employing more inexpensive RNA extraction kits. Another option for cost reduction is the HRM analysis. Open-source alternatives to commercial software are available (for example see [47]). The clustering algorithms of alternate software may rely on different parameters, and free versions probably require some manual effort to identify potential non-specific binding. Still, open-source software is a viable option to lower starting cost barriers, or to maximize customization, for studies using HRM analysis.

Gene expression is becoming a valuable tool for understanding the health of numerous species. With a greater understanding of how animal health and environmental pollutants affect the expression of specific genes (e.g. [10,48]), this information can be used to develop a ‘health panel’ used to track an individual's health parameters. Options to reduce costs, such as streamlining sex determination with the *XIST* method, will help to make health information more accessible. This is particularly ideal for long-term monitoring studies, as cost reduction options can help to conserve resources and extend datasets over longer periods of time.

## Data Availability

The data used in this study is available at UQ eSpace https://doi.org/10.48610/3f8d326. The data are also provided in the electronic supplementary material [49].
